# Trial sequential analysis of efficacy and safety of direct oral anticoagulants and vitamin K antagonists against left ventricular thrombus

**DOI:** 10.1038/s41598-023-40389-x

**Published:** 2023-08-14

**Authors:** Tetsuji Kitano, Yosuke Nabeshima, Masaharu Kataoka, Masaaki Takeuchi

**Affiliations:** 1 Second Department of Internal Medicine, University of Occupational and Environmental Health, School of Medicine, 1-1 Iseigaoka, Yahatanishi-ku, Kitakyushu, 807-8555 Japan; 2 Department of Laboratory and Transfusion Medicine, University of Occupational and Environmental Health Hospital, 1-1 Iseigaoka, Yahatanishi-ku, Kitakyushu, 807-8556 Japan

**Keywords:** Cardiology, Cardiovascular diseases, Platelets

## Abstract

Meta-analysis may increase the risk of random errors. Trial sequential analysis (TSA) has been developed to adjust for these random errors. We conducted TSA on the efficacy and safety of direct oral anticoagulants (DOACs) and vitamin K antagonists (VKAs) in left ventricular thrombus (LVT) patients in order to estimate how many additional patients should be required to draw definite conclusions. PubMed, Scopus, and Cochrane Library databases were searched for articles directly comparing DOACs and VKAs for LVT in LV *thrombus resolution*, *stroke*, *any thromboembolism*, *major bleeding*, *any bleeding*, and *all-cause death*. TSA was conducted with a cumulative Z-curve, monitoring boundaries, and required sample size. A simulated trial was run and TSA estimated the sample sizes of trials needed to draw definite conclusions. Of 4749 articles, 25 studies were used for the analysis. TSA revealed the current sample size already demonstrated superiority of DOACs in LV *thrombus resolution* and *stroke,* and futility in *any thromboembolism* and *all-cause death*. Two other outcomes did not achieve the required sample size. The sample size of new trials needed to demonstrate the superiority of DOACs over VKAs was estimated 400 for *any bleeding*. Corresponding trials needed to demonstrate no significant differences could be estimated for *major bleeding* and *any bleeding* (n = 200 and n = 2000, respectively). Current results show that the sample size required to draw definite conclusions was not reached for two outcomes, and there was a risk of random error. Further randomized controlled trials with sample sizes estimated by TSA will work effectively to obtain valid conclusions.

## Introduction

Direct oral anticoagulants (DOACs) are indicated for prevention of ischemic stroke and systemic embolism caused by non-valvular atrial fibrillation (AF), and for treatment and prevention of recurrent venous thromboembolisms (VTE); however, they have not been approved to treat left ventricular thrombus (LVT). Recently, several small, randomized controlled trials (RCTs)^1–3^ and many observational studies^4–24^ on off-label use of DOACs for LVT have been published, attracting attention to the potential use of DOACs in treatment of LVT. However, results regarding efficacy and safety of DOACs vary among reports. Although many meta-analyses comparing DOACs and vitamin K antagonists (VKAs) for LVT have been published in the past few years^25–34^, no definitive consensus has been reached yet, and some controversy remains. Most of these meta-analyses concluded that the efficacy and safety of DOACs should be evaluated in large RCTs, but implementation is a major challenge because the low incidence of LVT requires significant effort in patient recruitment and large RCTs require significant funding. Therefore, evidence from small RCTs and observational studies that have accumulated so far should be carefully assessed, and minimal RCTs should be conducted to complement evidence that is currently lacking.

Trial sequential analysis (TSA) is conceptually similar to sequential interim analysis of RCTs, in which trial results are validated at regular intervals to determine whether certain differences, or lack thereof, due to interventions, have been conclusively demonstrated^35^. TSA can provide a required sample size and can adjust boundaries for trial sequential monitoring for benefit, harm, and futility. Conclusions drawn using TSA demonstrate greater reliability than those relying upon traditional meta-analytical techniques^36^. Furthermore, TSA can estimate how many more patients will need to undergo RCT before performing meta-analyses can provide convincing and reliable conclusions^37^.

Accordingly, we conducted TSA to evaluate the current evidence on the efficacy and safety of DOACs and VKAs in patients with LVT, and to estimate how many additional patients should be enrolled in future trials to conclude benefit, harm, or futility from cumulative results of the meta-analysis.

## Methods

This study was conducted partly with secondary use of data in our previous systematic review and meta-analysis (CRD42021230849)^26^. Among the data in the previous publications, only those that directly compared the outcomes of DOACs and VKAs were used, excluding single-arm studies that investigated outcomes of either DOACs or VKAs. In addition, we searched again through February 9, 2023 to include articles that directly compared outcomes of DOACs and VKAs after our previous publication using the search terms “anticoagulant/vitamin K antagonist/warfarin”, “left ventricular/left ventricle/intraventricular”, and “thrombus/thrombi”. Details of “Search strategy”, “Article selection”, and “Data extraction” are described in Supplementary Table S1. A systematic review and meta-analysis were conducted according to PRISMA (Preferred Reporting Items for Systematic Reviews and Meta-Analyses) guidelines. Articles directly comparing VKAs and DOACs for at least one of the following outcomes in LVT patients: *thrombus resolution*, *stroke*, *any thromboembolism*, *major bleeding*, *any bleeding*, and *all-cause death* were extracted.

### Conventional meta-analysis

Pooled odds ratios (OR) and 95% confidence intervals (CI) were computed based on a random effects model and are presented as forest plots.

### TSA

TSA is a statistical approach that can determine sufficient sample sizes and reduce false inferences from meta-analyses due to type 1 or type 2 errors. This approach combines traditional meta-analysis with a method of calculating sample sizes and adjusting boundaries of sequential monitoring of trials for benefit, harm, and futility. In TSA, monitoring boundaries are applied and effect sizes are tested with each trial added to the analysis until the boundary of significance (benefit or harm) or lack of significance (futility) is achieved.

**Figure 1:**
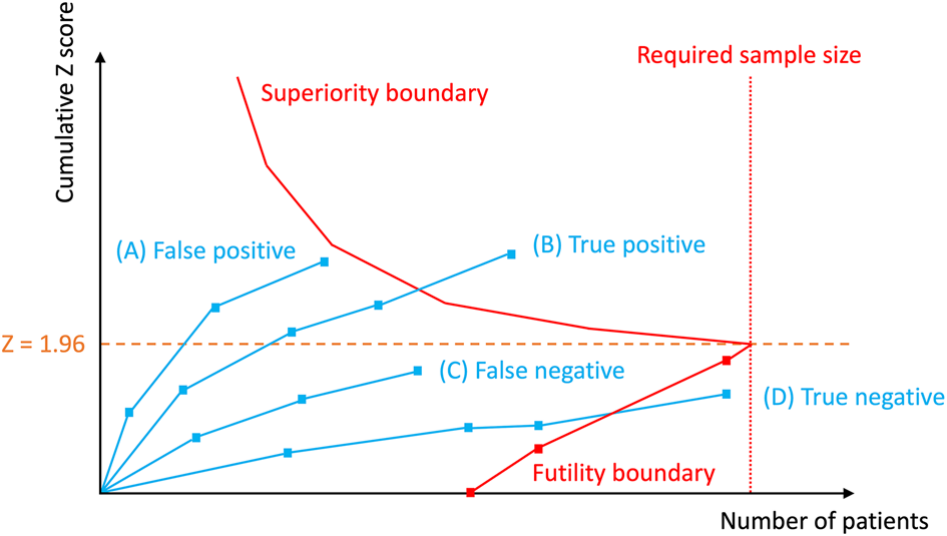
Schema of four examples of trial sequential analysis (TSA). (**A**) False positive: the cumulative Z-curve crosses the traditional boundary (Z =  ± 1.96, p = 0.05), but does not cross the superiority boundary. (**B**) True positive: the cumulative Z-curve crosses the superiority boundary before the required sample size is reached. (**C**) False negative: the cumulative Z-curve crosses neither the traditional boundary nor the futility boundary. (**D**) True negative: the required sample size is not reached but the cumulative Z-curve crosses the futility boundary.

If cumulative data (Z-curve) cross the traditional boundary (Z =  ± 1.96, p = 0.05), but do not cross the superiority boundary, an effect is considered false and the trial should be continued to detect or reject the effect of the intervention (false positive) (Fig. 1A). If the cumulative Z-curve crosses the superiority boundary before the required sample size is reached, it is considered firm evidence and no further trials are needed (true positive) (Fig. 1B). If the cumulative Z-curve does not cross the traditional boundary and does not cross the futility boundary, evidence is considered insufficient and the trial should be continued to detect or reject the effect of the intervention (false negative) (Fig. 1C). If the required sample size is not reached but the cumulative Z-curve crosses the futility boundary, it indicates no intervention effect and further trials are meaningless (true negative) (Fig. 1D).

### Statistical analysis

Continuous data are presented as means ± SDs or as medians and interquartile ranges, according to the data distribution. Categorical values are expressed as number and percentages. For conventional meta-analysis, statistical analyses were performed using R (version 4.2.1, The R foundation for Statistical Computing, Vienna). TSA was conducted using TSA software version 0.9.5.10 Beta (Copenhagen Trial Unit, Copenhagen, Denmark). Detailed instructions for TSA software can be found in the user manual^38^. The actual analytical procedure using TSA is described in Supplementary Fig. S1. A brief explanation of the main points is as follows. TSA was conducted to calculate the sample size required to demonstrate the risk difference with 80% power and a type 1 error of 5%. The risk difference was estimated based on overall results from previous publications: 6% for LV *thrombus resolution* (DOACs: 74%, VKAs: 68%), 3% for *stroke* (DOACs: 7%, VKAs: 10%), 5% for *any thromboembolism* (DOACs: 16%, VKAs: 21%), 2% for *major bleeding* (DOACs: 2%, VKAs: 4%), 2% for *any bleeding* (DOACs: 8%, VKAs: 10%), and 4% for *all-cause death* (DOACs: 6%, VKAs: 10%).

To estimate the number of trials needed to draw definitive conclusions about outcomes in the future, two simulated trials were set up and TSA was conducted. For superiority analysis, it is necessary for the cumulative Z-curve to cross the superiority boundary to draw a definitive conclusion. A simulated trial was set up in which the Z-curve crossed the superiority boundary, assuming that there were predefined risk differences between DOACs and VKAs. The risk difference between DOACs and VKAs for each outcome was assumed to be the aforementioned values, and the minimum sample size was calculated to cross the superiority boundary. For futility analysis, a simulated trial was set up in which the Z-curve crossed the futility boundary, assuming that there were no risk differences between DOACs and VKAs. The risks of LV *thrombus resolution*, *stroke*, *any thromboembolism*, *major bleeding*, *any bleeding*, and *all-cause death* were assumed to be 70%, 8%, 20%, 3%, 10%, and 8% for both DOACs and VKAs, respectively, and the minimum sample size was measured to cross the futility boundary. Quality assessment of the articles was conducted using the Newcastle–Ottawa Assessment Scale for observational studies and the Cochrane risk of bias tool for RCTs in meta-analyses^39,40^.

## Results

Articles directly comparing VKAs and DOACs for at least one of the outcomes in patients with LVT were selected based on PRISMA guidelines (Supplementary Fig. S2). Of the 4749 articles retrieved, 1129 were excluded due to duplication and 1117 due to publication before DOACs were commercially available. 2432 were excluded because their titles or abstracts did not meet inclusion criteria, and 71 were read in full text. 46 articles that met one or more of the exclusion criteria were excluded after reading the full text. Finally, 25 articles that directly compared DOACs and VKAs were included in this meta-analysis. Of 3490 patients, 1047 treated with DOACs and 2443 treated with VKAs. These publications included three RCTs, 20 retrospective observational studies, and two prospective observational studies. Tables 1 and 2 show a summary of included articles, the main clinical characteristics, and comparison between DOACs and VKAs. Qualitative assessment of the articles is presented in Supplementary Table S2 and S3.

**Table 1:** Summary of included articles.

First author	Year	Study design	Number (DOAC/VKA)	Age (DOAC/VKA)	Male (DOAC/VKA)	Etiology	F/U (months)	Outcome	Event (DOAC/VKA)	DOAC type
McCarthy	2019	Retro OBS	4/94	NA	NA	HF, MI	12	Resolution	4/71	Apixaban 3 Rivaroxaban 1
Ali	2020	Retro OBS	32/60	59 ± 12/ 58 ± 6	26/49	ICM, NICM, MI, TCM	12	Resolution	18/37	Apixaban 13 Dabigatran 1 Rivaroxaban 18
							12	Stroke	2/7	
							12	Any thromboembolism	2/14	
Cochrane	2020	Retro OBS	14/59	59 ± 30/ 60 ± 37	11/45	CAD, HF, Arrhythmia	12	Resolution	12/45	NA
							12	Stroke	0/9	
							12	Any bleeding	2/8	
							12	All-cause death	1/2	
Daher	2020	Retro OBS	17/42	57 ± 14/ 61 ± 13	14/35	MI, ICM, HF, VA	12	Resolution	12/30	Apixaban 12 Dabigatran 1 Rivaroxaban 4
							3	Any thromboembolism	2/4	
Guddeti	2020	Retro OBS	19/80	61 ± 13/ 31 ± 12	15/55	ICM, NICM	12	Stroke	0/2	Apixaban 15 Dabigatran 2 Rivaroxaban 2
							12	Any thromboembolism	0/2	
							12	Any bleeding	1/4	
Iqbal	2020	Retro OBS	22/62	62 ± 13/ 62 ± 14	20/55	ICM, DCM, HCM, Myocarditis	8	Resolution	13/42	Apixaban 8 Dabigatran 1 Rivaroxaban 13
							36	Stroke	0/1	
							36	Any thromboembolism	0/2	
							36	Major bleeding	0/6	
							36	Any bleeding	0/6	
							36	All-cause death	3/6	
Jones	2020	Pros OBS	41/60	59 ± 14/ 61 ± 14	33/51	MI	24	Resolution	34/38	Apixaban 15 Edoxaban 2 Rivaroxaban 24
							26.4	Stroke	1/3	
							26.4	Any thromboembolism	1/3	
							26.4	Major bleeding	0/7	
							26.4	Any bleeding	6/22	
Ratnayake	2020	Retro OBS	2/42	NA	NA	MI	6	Resolution	1/34	Dabigatran 2
Robinson	2020	Retro OBS	121/236	58 ± 15/ 58 ± 15	94/170	ICM, NICM, HCM	11.5	Any thromboembolism	17/14	NA
							11.5	Any bleeding	8/19	
							11.5	All-cause death	14/32	
Willeford	2020	Retro OBS	22/129	56 ± 12/ 58 ± 13	17/104	NA	5.7	Resolution	13/63	Apixaban 4 Rivaroxaban 18
							5.7	Stroke	0/7	
							5.7	Any thromboembolism	0/8	
							5.7	Major bleeding	1/5	
							5.7	Any bleeding	1/5	
Abdelnabi	2021	RCT	39/40	49 ± 12/ 50 ± 13	21/24	ICM, Idiopathic CM	6	Resolution	34/32	Rivaroxaban 39
							6	Stroke	0/4	
							6	Any thromboembolism	0/6	
							6	Major bleeding	2/6	
Albabtain	2021	Retro OBS	28/35	58 ± 18/ 59 ± 16	24/34	MI, HF,	22	Resolution	20/24	Rivaroxaban 28
							22	Stroke	1/1	
							22	Any bleeding	2/1	
							22	All-cause death	0/0	
Alcalai	2021	RCT	18/17	56 ± 13/ 59 ± 10	13/15	MI	3	Resolution	17/16	Apixaban 18
							NA	Stroke	0/1	
							NA	Any thromboembolism	0/1	
							NA	Any bleeding	0/2	
							NA	All-cause death	1/0	
Bass	2021	Retro OBS	180/769	63 ± 17/ 62 ± 15	125/545	NA	3	Stroke	14/90	Apixaban 79 Dabigatran 27 Rivaroxaban 74
							3	Any thromboembolism	55/254	
							3	Major bleeding	3/22	
							3	Any bleeding	14/84	
Hofer	2021	Pros OBS	10/33	NA	NA	NSTEMI, MI	14	Resolution	7/20	NA
Isa	2021	RCT	14/13	55 ± 11/ 55 ± 11	13/12	HF	NA	Stroke	1/0	Apixaban 14
							NA	Major bleeding	0/1	
							NA	All-cause death	2/4	
Iskaros	2021	Retro OBS	32/45	62/ 63	28/41	NA	6	Resolution	27/34	Apixaban 24 Dabigatran 1 Rivaroxaban 7
							3	Stroke	1/0	
							3	Any thromboembolism	1/0	
							3	Any bleeding	1/2	
							3	All-cause death	0/0	
Mihm	2021	Retro OBS	33/75	63 ± 14/ 60 ± 14	23/54	Various cardiac disease	NA	Resolution	14/26	Apixaban 23 Rivaroxaban 10
							NA	Stroke	2/4	
							NA	Any thromboembolism	3/4	
							NA	Major bleeding	5/2	
							NA	All-cause death	4/6	
Varwani	2021	Retro OBS	36/25	NA	NA	NA	NA	Resolution	20/16	Apixaban NA Dabigatran NA Rivaroxaban NA
							NA	Stroke	1/1	
							NA	Any bleeding	3/2	
Xu	2021	Retro OBS	25/62	59 ± 12/ 62 ± 12	6/15	ICM, MI, HCM	27	Resolution	19/46	Dabigatran NA Rivaroxaban NA
							27	Stroke	1/3	
							27	Any thromboembolism	1/4	
							27	Any bleeding	1/2	
							27	All-cause death	2/3	
Zhang	2021	Retro OBS	33/31	60 ± 15/ 61 ± 9	24/23	MI	24	Resolution	26/23	Rivaroxaban 33
							NA	Any thromboembolism	1/4	
							NA	Major bleeding	0/1	
							NA	Any bleeding	2/3	
							NA	All-cause death	1/4	
Herald	2022	Retro OBS	134/299	NA	NA	NA	41	Stroke	26/73	Apixaban 20 Dabigatran 108 Rivaroxaban 6
							41	Any thromboembolism	29/84	
							41	Any bleeding	37/113	
							41	All-cause death	32/138	
Rahunathan	2022	Retro OBS	14/4	59 ± 13/ 64 ± 5	12/3	ICM, DCM, Myocarditis	4.7	Resolution	6/1	NA
							4.7	Stroke	0/0	
							4.7	Any thromboembolism	0/0	
							4.7	Major bleeding	0/0	
Zhang	2022	Retro OBS	109/78	65/ 63	85/66	HF	17	Resolution	77/46	Rivaroxaban NA
							17	Any thromboembolism	5/10	
							17	Major bleeding	0/1	
							17	Any bleeding	8/5	
							17	All-cause death	31/27	
Seiler	2023	Retro OBS	48/53	64 ± 12/ 62 ± 14	42/41	ICM, Idiopathic CM, HCM, TCM, Myocarditis, Tachy CM, Chemo-induced CM	26.6	Resolution	40/37	Apixaban 17 Rivaroxaban 31
							26.6	Stroke	4/4	
							26.6	Any thromboembolism	6/5	
							26.6	Major bleeding	3/2	
							26.6	Any bleeding	5/9	
							26.6	All-cause death	4/6	

*CAD* coronary artery disease, *CM* cardiomyopathy, *D* direct oral anticoagulant, *DCM* dilatated cardiomyopathy, *DOAC* direct oral anticoagulant, *F/U* follow-up duration, *HCM* hypertrophic cardiomyopathy, *HF* heart failure, *ICM* ischemic cardiomyopathy, *MI* myocardial infarction, *NA* not applicable, *NICM* non-ischemic cardiomyopathy; , *NSTEMI* non-ST elevated myocardial infarction, *OBS* observational study, *Pros* prospective, *RCT* randomized control trail, *Retro* retrospective, *TCM* takotsubo cardiomyopathy, *V* vitamin k antagonist, *VA* ventricular arrhythmia.

**Table 2:** Clinical characteristics and the comparison between treatment groups.

Parameters	VKAs (n = 2443)		DOACs (n = 1047)		p value
	Number	Measurements	Number	Measurements	
Age	1905	60.5 ± 14.3	829	60.1 ± 14.6	0.504
Male	1950	1437 (74%)	861	646 (75%)	0.456
HT	1017	561 (55%)	603	332 (55%)	0.968
DM	1136	365 (32%)	649	230 (35%)	0.154
DL	506	222 (44%)	302	154 (51%)	0.050
CKD	1189	348 (29%)	385	86 (22%)	0.008
CAD	1128	669 (59%)	358	216 (60%)	0.730
AFib	1732	548 (32%)	732	209 (29%)	0.129
Smoking	567	238 (42%)	413	153 (37%)	0.120

Data are expressed as number (%) or means ± standard deviations.

*AFib* atrial fibrillation, *CAD* coronary artery disease, *CKD* chronic kidney disease, *DL* dyslipidemia, *DM* diabetes mellitus, *DOAC* direct oral anticoagulant, *HT* hypertension, *VKA* vitamin k antagonist.

### Left ventricular thrombus resolution

**Figure 2:**
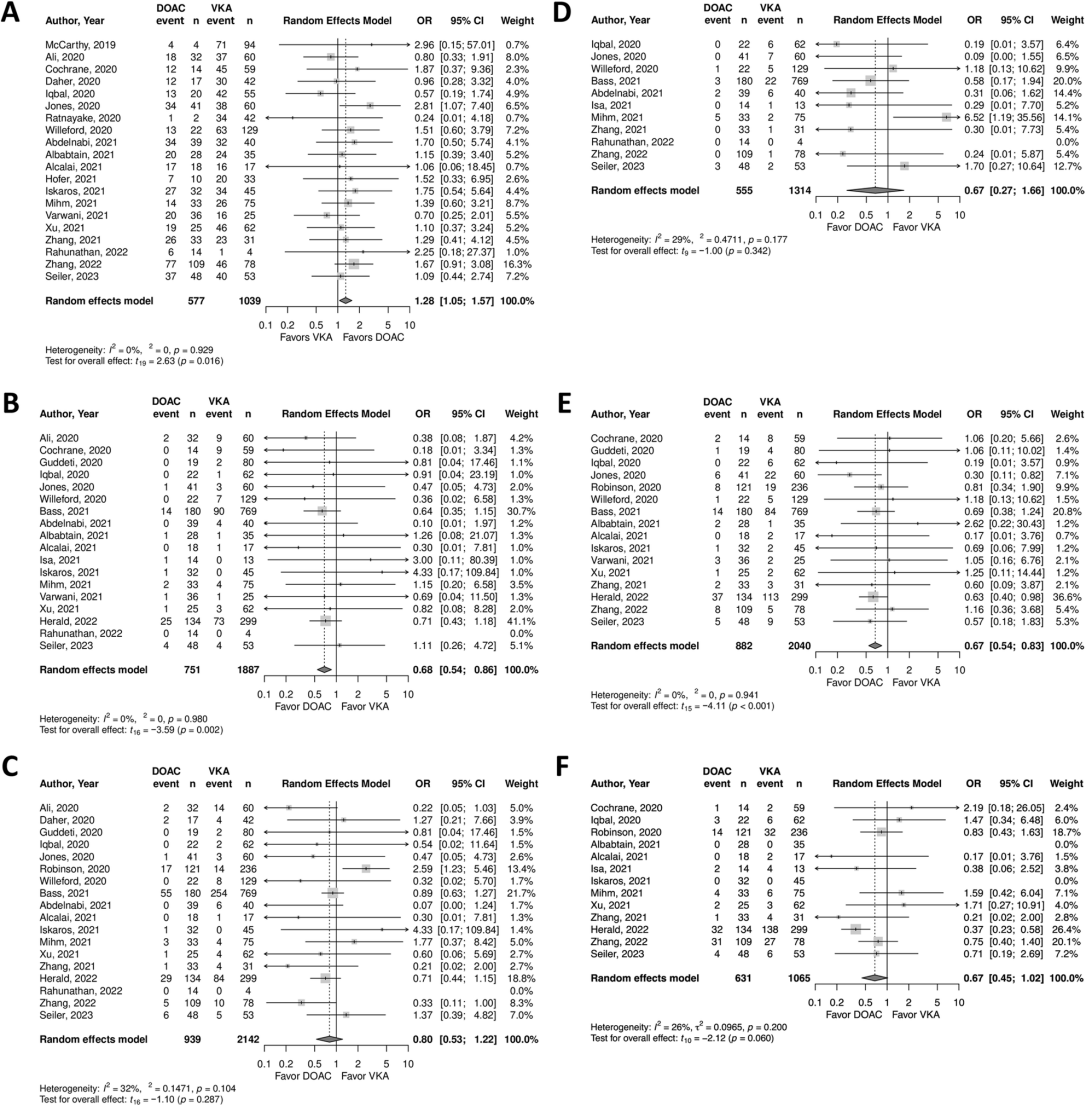
Forest plot of direct comparison in six outcomes for direct oral anticoagulants (DOACs) and vitamin K antagonists (VKAs). (**A**) Left ventricular thrombus resolution, (**B**) stroke, (**C**) any thromboembolism, (**D**) major bleeding, (**E**) any bleeding, (**F**) all-cause death. *CI* confidence interval, *DOACs* direct oral anticoagulants, *OR* odds ratio, *VKAs* vitamin K antagonists.

**Figure 3:**
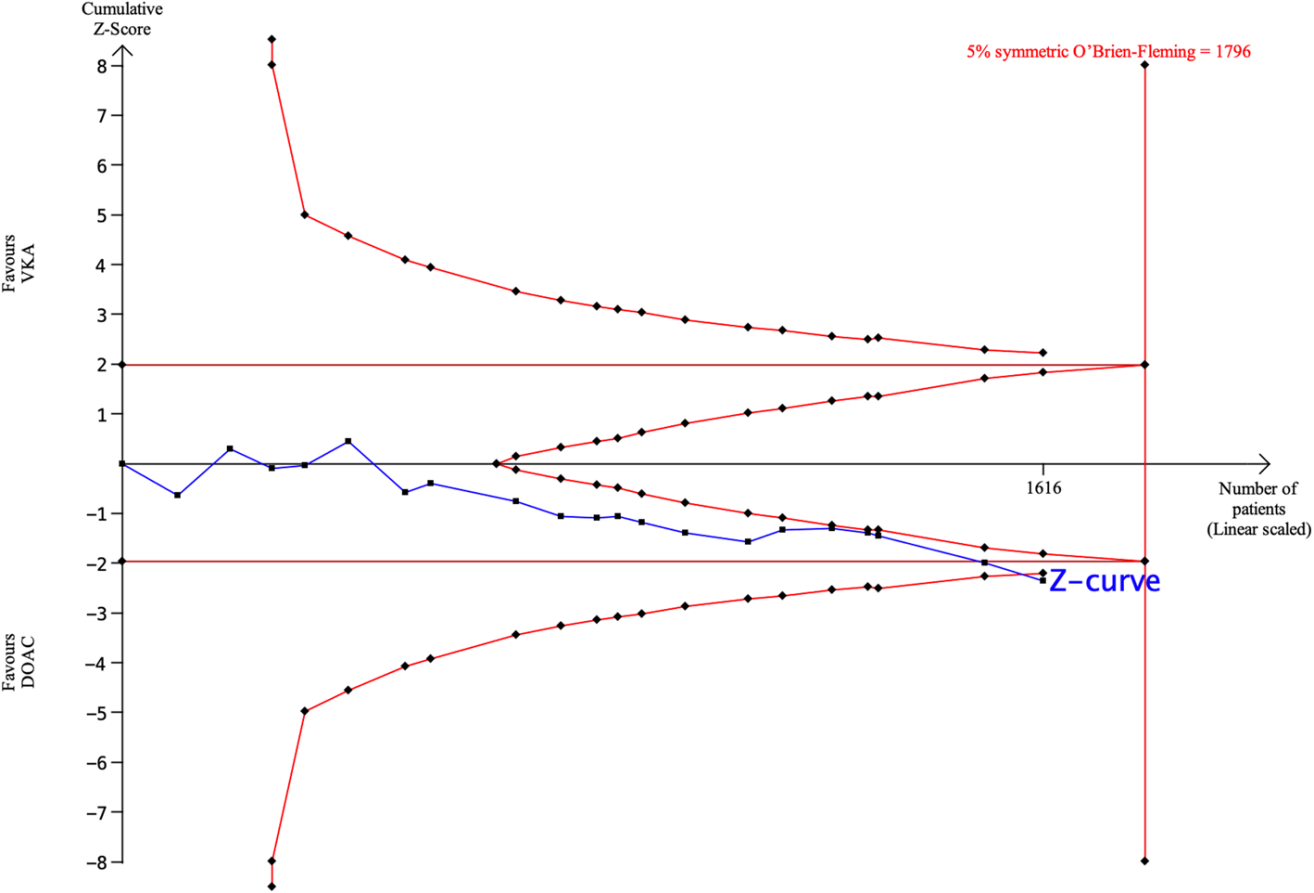
TSA of DOACs and VKAs in left ventricular (LV) *thrombus resolution* using power of 80%, 5% significance, to detect a 6% absolute difference (DOACs: 74%, VKAs: 68%). The required sample size was 1796 (vertical red line). The cumulative Z-curve (blue line with small black squares representing each trial) crossed both the traditional boundary (horizontal red line) and the superiority boundary (trial sequential monitoring boundary) (concave red line), indicating evidence is sufficient (true positive).

Twenty articles (2 RCTs and 18 observational studies) reported LV *thrombus resolution*. Conventional meta-analysis showed that the LV *thrombus resolution* rate was significantly higher with DOACs than with VKAs (pooled OR: 1.28, 95% CI 1.05–1.57, p = 0.02, Fig. 2A). Using TSA, the required sample size for significant or non-significant differences in LV *thrombus resolution* based on an event rate of 74% with DOACs and 68% with VKAs was 1796 patients (Fig. 3). The number of patients included in the present 20 trials was 1616, which indicated that 90% of the required number of patients have been included so far. Ninety percent of the required sample size was accumulated and the cumulative Z-curve crossed both the traditional boundary and the superiority boundary. Thus, the evidence from the pooled meta-analysis is considered conclusive and truly positive. Based on these results, it is concluded that DOACs have a significantly higher incidence of LV *thrombus resolution* than VKAs and the sample size is sufficient to demonstrate significance, indicating that further accumulation of evidence is unnecessary.

### Stroke

**Figure 4:**
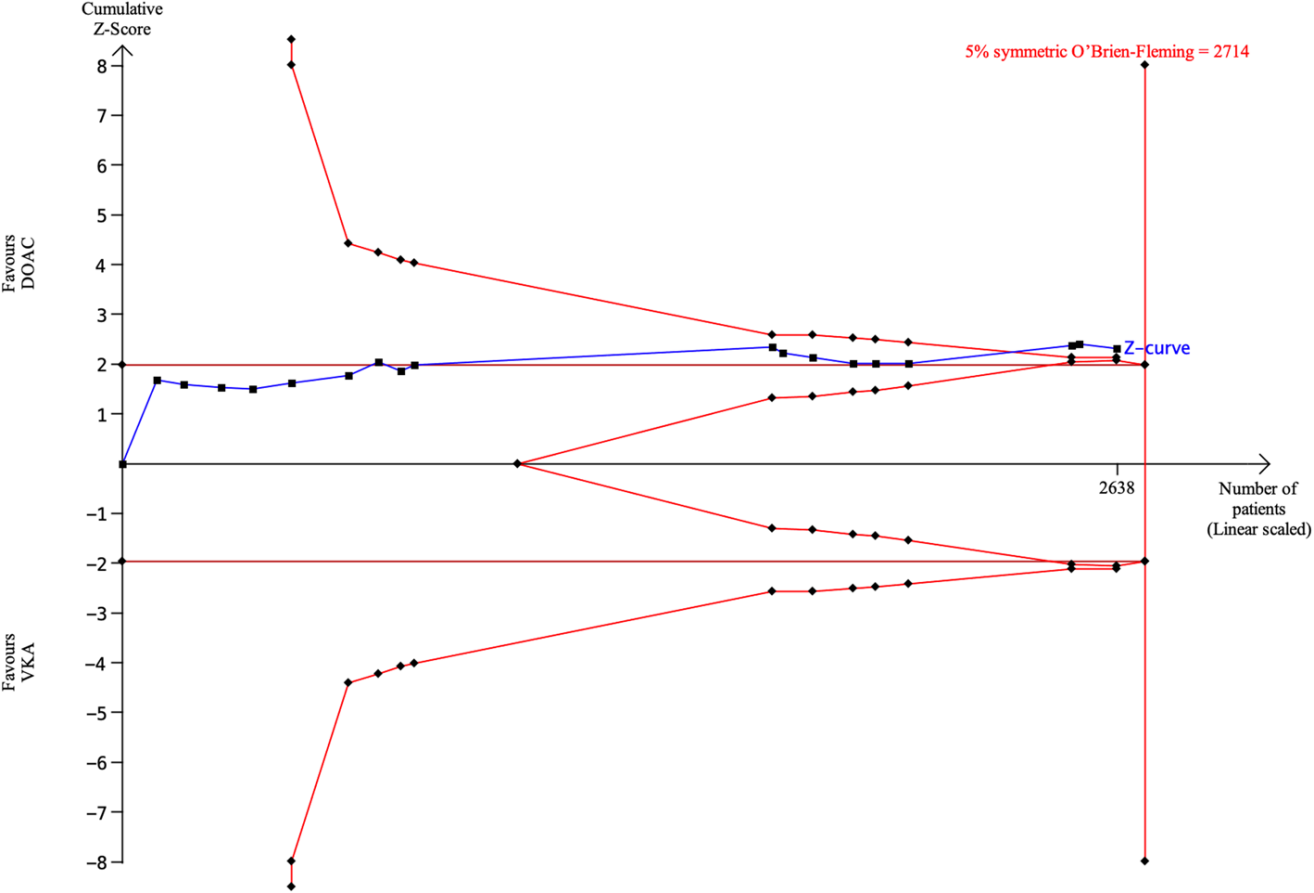
TSA of DOACs and VKAs in *stroke* using power of 80%, 5% significance, to detect a 3% absolute difference (DOACs: 7%, VKAs: 10%). The required sample size was 2714 (vertical red line). The cumulative Z-curve (blue line with small black squares representing each trial) crossed both the traditional boundary (horizontal red line) and the superiority boundary (trial sequential monitoring boundary) (concave red line), indicating evidence is sufficient (true positive).

Eighteen articles (3 RCTs and 15 observational studies) reported the *stroke* rate. Conventional meta-analysis showed that the incidence of *stroke* was significantly lower for DOACs than for VKAs (pooled OR 0.68, 95% CI 0.54–0.86, p < 0.01, Fig. 2B). Using TSA, the required sample size was 2714 patients (the number of patients in 18 articles was 2638) to demonstrate or reject the difference in incidence of stroke of 7% for DOACs and 10% for VKAs (Fig. 4). Ninety-seven percent of the required sample size was accumulated and the cumulative Z-curve crossed both the traditional boundary and the superiority boundary.

### Any thromboembolism

**Figure 5:**
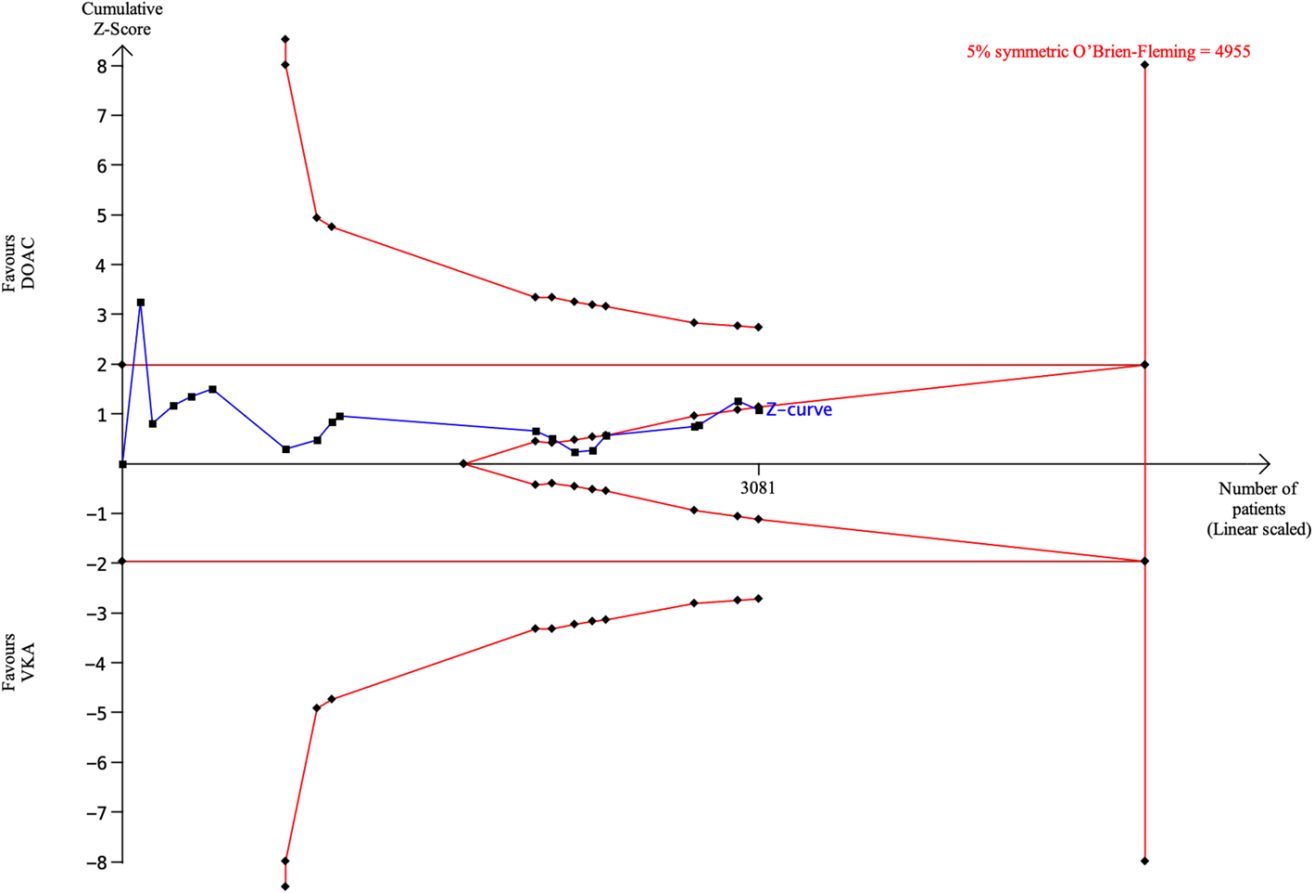
TSA of DOACs and VKAs in *any thromboembolism* using power of 80%, 5% significance, to detect a 5% absolute difference (DOACs: 16%, VKAs: 21%). The required sample size was 4955 (vertical red line). The cumulative Z-curve (blue line with small black squares representing each trial) crossed the futility boundary (convex red line), indicating evidence is sufficient (true negative).

Eighteen articles (2 RCTs and 16 observational studies) provided rates of *any thromboembolism*. A conventional meta-analysis showed that the incidence of *any thromboembolism* was comparable between DOACs and VKAs (pooled OR 0.80, 95% CI 0.53–1.22, p = 0.29, Fig. 2C). With TSA, the sample size required to demonstrate or reject the difference in the event rate of *any thromboembolism* of 16% for DOACs and 21% for VKAs was 4955 patients (Fig. 5). Currently, 3081 patients (62% of required sample size) are included and the cumulative Z-curve crossed the futility boundary. Therefore, the pooled meta-analysis was considered definitive with sufficient evidence (true negative). These results indicated that the incidence of *any thromboembolism* with DOACs does not differ significantly from that with VKAs and the sample size is sufficient to demonstrate futility, suggesting that further evidence accumulation is not warranted.

### Major bleeding

**Figure 6:**
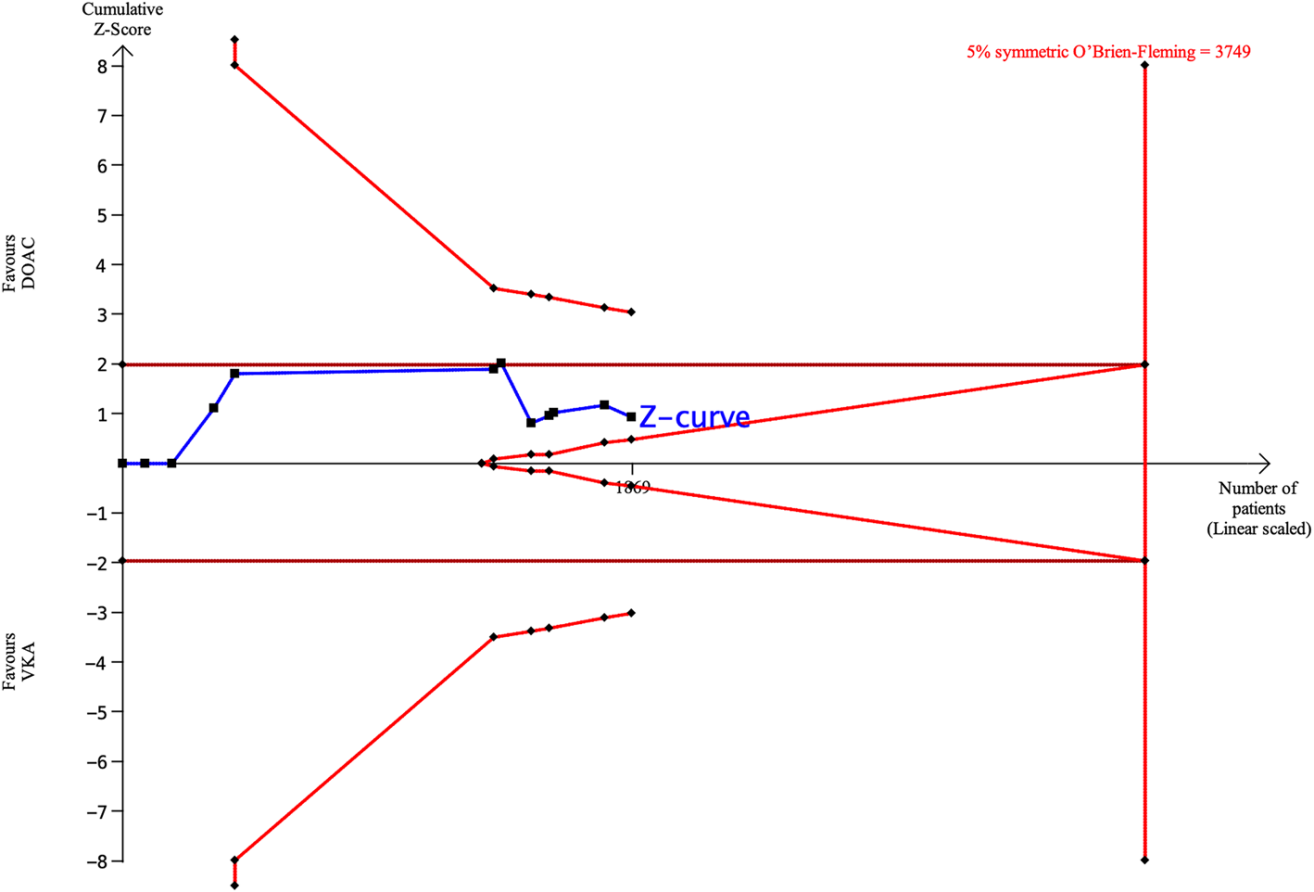
TSA of DOACs and VKAs in *major bleeding* using power of 80%, and 5% significance, to detect a 2% absolute difference (DOACs:2%, VKAs: 4%). The required sample size was 3749 (vertical red line). The cumulative Z-curve (blue line with small black squares representing each trial) did not cross the traditional boundary (horizontal red line), the superiority boundary (trial sequential monitoring boundary) (concave red line), or the futility boundary (convex red line), indicating a lack of evidence (false negative).

Eleven publications (2 RCTs and 9 observational studies) reported rates of *major bleeding*. A meta-analysis showed no significant difference between DOACs and VKAs in incidence of *major bleeding* (pooled OR 0.67, 95% CI 0.27–1.66, p = 0.34, Fig. 2D). To demonstrate or reject the difference in the event rate of *major bleeding* of 2% for DOACs and 4% for VKAs, a sample size of 3749 patients (versus only 1869 patients in the 11 studies) was estimated by TSA (Fig. 6). Fifty percent of the required sample size is available and the cumulative Z-curve did not cross either the traditional or futility boundary, suggesting a lack of evidence (false negative).

Assuming an event rate of 2% for DOACs and 4% for VKAs, the cumulative Z-curve could not cross the superiority boundary no matter how many patients were added. When 24,000 patients were added, the boundary of futility was crossed before the superiority or traditional boundaries were crossed (Supplementary Fig S3). Assuming an event rate of 3% for both DOACs and VKAs, 200 more patients would be required to cross the futility boundary (Supplementary Fig. S4).

### Any bleeding

**Figure 7:**
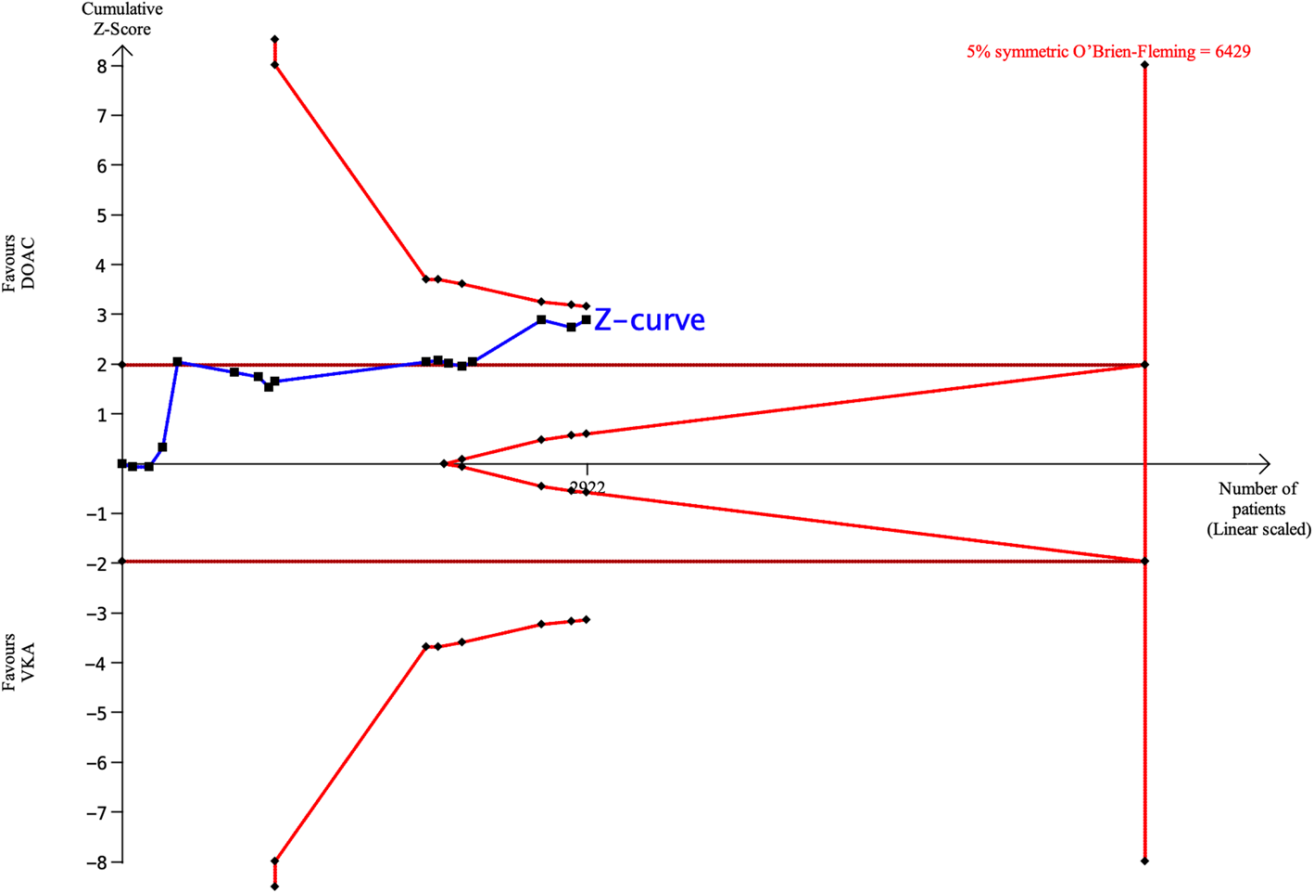
TSA of DOACs and VKAs in *any bleeding* using power of 80% and 5% significance, to detect a 2% absolute difference (DOACs: 8%, VKAs: 10%). The required sample size was 6429 (vertical red line). The cumulative Z-curve (blue line with small black squares representing each trial) crossed the traditional boundary (horizontal red line), but did not cross the superiority boundary (trial sequential monitoring boundary) (concave red line), indicating a lack of evidence (false positive).

There were 16 publications (1 RCT and 15 observational studies) that reported rates of *any bleeding*. The incidence of *any bleeding* was significantly lower for DOACs than for VKAs (pooled OR 0.67, 95% CI 0.54–0.83, p < 0.01, Fig. 2E). According to TSA, the sample size required to demonstrate or reject the difference in the incidence of *any bleeding* with an event rate of 8% for DOACs and 10% for VKAs was 6429 patients (Fig. 7). Only 2922 patients (45% of the required sample size) have been evaluated and the cumulative Z-curve crossed the traditional boundary, but did not cross the superiority boundary. Accordingly, the pooled meta-analysis evidence is not conclusive and is considered a spurious effect (false positive).

With an 8% event rate for DOACs and 10% event rate for VKAs, an additional 400 patients will need to be included to cross the superiority boundary (Supplementary Fig. S5). With an event rate of 10% for both DOACs and VKAs, 2000 patients would be required to cross the futility boundary (Supplementary Fig. S6).

### All-cause death

**Figure 8:**
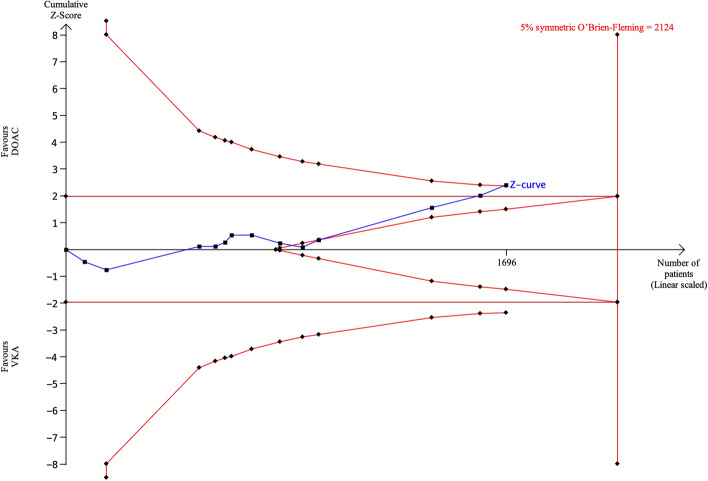
TSA of DOACs and VKAs in *all-cause death* using power of 80% and 5% significance, to detect a 4% absolute difference (DOACs:6%, VKAs: 10%). The required sample size was 2124 (vertical red line). The cumulative Z-curve (blue line with small black squares representing each trial) crossed both the traditional boundary (horizontal red line) and the superiority boundary (trial sequential monitoring boundary) (concave red lines), indicating evidence is sufficient (true positive).

Thirteen articles (2 RCTs and 11 observational studies) reported rates of *all-cause death*. The incidence of *all-cause death* did not differ significantly between DOACs and VKAs in a pooled analysis (pooled OR 0.67, 95% CI 0.45–1.02, p = 0.06, Fig. 2F). For TSA, the sample size required to determine whether there is a significant difference in *all-cause death* was 2124 patients, based on an event rate of 6% for DOACs and 10% for VKAs (Fig. 8). Eighty percent of the required sample size (1696 patients) was included and the cumulative Z-curve crossed the futility boundary. Accordingly, the pooled meta-analysis evidence was considered sufficient and definitive (true negative). These results supported the conclusion that the incidence of *all-cause death* with DOACs was not significantly different from that with VKAs, and the sample size was sufficient to prove futility, suggesting that no further accumulation of evidence is needed.

## Discussion

To the best of our knowledge, this is the first study to employ TSA regarding the efficacy and safety of DOACs and VKAs in LVT, and to determine sample sizes required to reach definitive conclusions for each outcome. The main findings of this study are summarized as follows: (1) DOACs and VKAs have been directly compared in three RCTs and 22 prospective or retrospective observational studies; (2) a conventional meta-analysis found that DOACs were favorable over VKAs for LV *thrombus resolution*, *stroke* and *any bleeding*, while *any thromboembolism*, *major bleeding*, and *all-cause death* were not significantly different in the two treatments; (3) TSA results showed that the superiority or futility boundary was reached for LV *thrombus resolution* and *stroke*, and *any thromboembolism* and *all-cause death*, whereas cumulative numbers of patients did not reach the required sample size for *major bleeding* and *any bleeding*, resulting in either type 1 or type 2 errors; (4) sample sizes (patient numbers) in future trials needed to demonstrate the superiority of DOACs could be estimated for *any bleeding*; (5) it is possible to estimate patient numbers needed in future trials to demonstrate that there are no significant differences between DOACs and VKAs for *major bleeding* and *any bleeding*.

### Previous studies

DOACs are convenient for daily clinical use because they do not require blood international normalized ratio monitoring or dietary restrictions and have a rapid onset of efficacy^41^. Furthermore, DOACs have proven useful in a variety of situations, such as cancer-related VTE^42^ and AF with bioprosthetic mitral valves^43^. Although LVT has traditionally been treated with VKAs, off-label use of DOACs has been increasingly reported during the last decade. Several meta-analyses compared efficacy and safety of DOACs and VKAs for LVT, but conclusions conflicted among publications^25–34^.

Although meta-analyses are considered the highest level of evidence for assessing benefits and harms of interventions, and while they permit strong inferences about the quality of available evidence, they may also increase the risk of random errors (type 1 error [false positive] or type 2 error [false negative]) when statistical tests are performed repetitively on accumulated data, or when sample sizes or numbers of events are insufficient^44–47^. Meta-analyses that do not reach the required sample size increase the risk of over- or underestimating effects of treatment, leading to spurious inferences^48^. To overcome these problems, trial sequential analysis (TSA) has been developed to adjust for these random errors and to address false conclusions^38^.

### Current study

In the current study, which included three RCTs and 22 observational studies, a random-effects model showed no significant differences between DOACs and VKAs for *any thromboembolism*, *major bleeding*, and *all-cause death*, while the incidence of LV *thrombus resolution*, *stroke*, and *any bleeding* differed significantly. However, when performing TSA, the sample size required to draw definitive conclusions was not reached for two of the six outcomes (*major bleeding* and *any bleeding*). Although *major bleeding* showed no significant difference between DOACs and VKAs in the conventional meta-analysis, TSA showed false-negative and no definitive conclusions were reached. The same was true for *any bleeding* showed significantly lower incidence among patients with taking DOACs in the conventional meta-analysis, whereas TSA showed a false-positive, which did not lead to a definitive conclusion. TSA revealed that the results of conventional meta-analyses for these two outcomes lack sufficient evidence and that it would be misleading to draw conclusions based only upon conventional meta-analysis results. On the other hand, regarding LV *thrombus resolution*, *stroke*, *any thromboembolism*, and *all-cause death*, the cumulative Z-curve crossed the superiority or futility boundary, and sufficient sample size for a definitive conclusion was reached.

For *major bleeding* and *any bleeding*, simulation trials were used to determine additional sample sizes required. Assuming that event rates are the same for DOACs and VKAs in simulated trials, we determined the additional sample sizes necessary to draw conclusions that there are no significant differences between the two groups. In contrast, when assuming that the DOACs is preferable to VKAs, it was possible to determine the number of additional samples needed to conclude the superiority of DOACs, except for *major bleeding*. For *major bleeding*, no matter how many trials were added to show the superiority of the DOACs, the required number of samples did not cross the superiority boundary before crossing the futility boundary.

As far as we know, several RCTs comparing DOACs and VKAs for LVT are currently ongoing (NCT04970576; NCT05705089). A trial evaluated LV *thrombus resolution*, *stroke* or *systemic embolism*, and *major bleeding* in LVT with acute coronary syndromes treated with Rivaroxaban or Warfarin (NCT04970576). Given the results of our TSA, an additional 200 patients were needed to demonstrate that there was no difference between DOACs and VKAs in *major bleeding*.

### Study limitations

The limitations of this study include the following. First, almost all limitations of meta-analysis also apply to TSA, because TSA uses data from meta-analyses. Publication bias and risk of bias in individual studies may affect the results of TSA. Second, since there are only three RCTs comparing DOACs and VKAs for LVT, the present TSA included observational studies in addition to RCTs. Although TSA is generally used to analyze RCTs, our study also included observational studies. The quality of each observational study was scored using the Newcastle–Ottawa Assessment Scale, and the quality of most studies was high; however, results need to be interpreted with caution. Third, the degree of bleeding and the proportion of time in the therapeutic range for VKAs have not been standardized among studies, and some patients also received concomitant antiplatelet agents, which may have affected the results. Fourth, follow-up duration in each event varied among the included studies, which may have affected the results. Fifth, in TSA and in estimating future needed sample sizes, predefined event rates for DOACs and VKAs were used for each calculation, and some of these assumptions may not be correct.

## Conclusions

TSA revealed that the current sample sizes have still not been reached to draw definite conclusions for *major bleeding* and *any bleeding*, and there is a risk of random error. TSA is a useful method to estimate sample sizes of further RCTs to draw definitive conclusions.

### Supplementary Information

Supplementary Information.

## Data Availability

All datasets analyzed in this study are available from the corresponding author on reasonable request.
