# Supplementary material for: Trial sequential analysis of efficacy and safety of direct oral anticoagulants and vitamin K antagonists against left ventricular thrombus
Source: Sci Rep. 2023 Aug 14;13:13203. doi: 10.1038/s41598-023-40389-x (PMC10425444; doi:10.1038/s41598-023-40389-x)
Supplement: Supplementary file 1 — Supplementary Information. [file 41598_2023_40389_MOESM1_ESM.docx]

**Supplementary file**

**Trial sequential analysis of efficacy and safety of direct oral anticoagulants and vitamin K antagonists against left ventricular thrombus**

**Address for correspondence: Tetsuji Kitano, MD, PhD**

**Supplemental Table S1: Methods on “search strategies”, “article selection”, and “data extraction”**

**Search strategies**

**Cochrane library**

**Search date on 9/February/2023**

1. **Cochrane library:**

1: (anticoagulant OR (vitamin k antagonist) OR warfarin): ti, ab, kw

2: ((left ventricular) OR (left ventricle) OR intraventricular): ti, ab, kw

3: (thrombus OR thrombi): ti, ab, kw

3: #1 AND #2 AND #3

Results: 131 articles

**Scopus**

**Search date on 9/February/2023**

1. **Scopus:**

1: TITLE-ABS-KEY (anticoagulant OR (vitamin AND k AND antagonist) OR warfarin)

2: TITLE-ABS-KEY ((left AND ventricular) OR (left AND ventricle) OR intraventricular)

3: TITLE-ABS-KEY (thrombus OR thrombi)

4: #1 AND #2 AND #3

Results: 2,354 articles

**PubMed**

**Search date on 9/February/2023**

1. **PubMed:**

Keywords: Search (anticoagulant OR (vitamin k antagonist) OR warfarin) AND ((left ventricular) OR (left ventricle) OR intraventricular) AND (thrombus OR thrombi)

Results: 2,263 articles

**Records After Duplicates Removed n=3,620**

**Article selection**

| Inclusion criteria | (1) Patients with left ventricular thrombus (LVT) who were treated with vitamin K antagonists (VKAs) or direct oral anticoagulants (DOACs) |
| --- | --- |
|  | (2) Articles reporting at least one of the following outcomes: *thrombus resolution*, *stroke*, *any thromboembolism*, *major bleeding*, *any bleeding*, or *all-cause death* |
|  | (3) Articles reporting information on both VKAs and DOACs treatment, including numbers of patients and events |
|  | (4) Articles on human adults |
|  | (5) Articles published after 2009 |
| Exclusion criteria | (1) Articles that included intracardiac thrombus only in locations other than the left ventricle |
|  | (2) Articles that included only patients <18 years old or animals |
|  | (3) Articles that included fewer than 10 eligible patients |
|  | (4) Case reports, case series, or abstracts |

**Data extraction**

| Collected data |
| --- |
| (1) first author’s name |
| (2) year of publication |
| (3) number of study patients |
| (4) number of events |
| (5) mean or median age |
| (6) gender distribution |
| (7) type of treatment |
| (8) incidence of events, including *thrombus resolution*, *stroke*, *any embolism*, *major bleeding*, *any bleeding*, *all-cause death* |
| (9) time to event |
| (10) type of DOACs (if stated) |
| (11) etiology of heart diseases |
| (12) risk factors |

**Supplemental Table S2: Quality assessment of included observational studies using the Newcastle Ottawa Scale.**

| Author_Publication year | Selection | | | | Comparability | Outcome | | | Total |
| --- | --- | --- | --- | --- | --- | --- | --- | --- | --- |
| McCarthy_2019 | ★ | ★ | ★ | ★ | - | ★ | ★ | ★ | 7 |
| Ali_2020 | ★ | ★ | ★ | ★ | ★ | ★ | ★ | ★ | 8 |
| Cochrane_2020 | ★ | ★ | ★ | ★ | ★ | ★ | ★ | ★ | 8 |
| Daher_2020 | ★ | ★ | ★ | ★ | ★ | ★ | - | ★ | 7 |
| Guddeti_2020 | ★ | ★ | ★ | ★ | ★ | ★ | ★ | ★ | 8 |
| Iqbal_2020 | ★ | ★ | ★ | ★ | ★ | ★ | ★ | ★ | 8 |
| Jones_2020 | ★ | ★ | ★ | ★ | ★ | ★ | ★ | ★ | 8 |
| Ratnayake_2020 | ★ | ★ | ★ | ★ | - | ★ | ★ | ★ | 7 |
| Robinson_2020 | ★ | ★ | ★ | ★ | ★★ | ★ | ★ | ★ | 8 |
| Willeford_2020 | ★ | ★ | ★ | ★ | ★★ | ★ | ★ | ★ | 9 |
| Albabtain_2021 | ★ | ★ | ★ | ★ | ★ | ★ | ★ | ★ | 8 |
| Bass_2021 | ★ | ★ | ★ | ★ | ★★ | ★ | - | ★ | 8 |
| Hofer_2021 | ★ | ★ | ★ | ★ | ★ | ★ | ★ | ★ | 8 |
| Iskaros_2021 | ★ | ★ | ★ | ★ | ★ | ★ | ★ | - | 7 |
| Mihm_2021 | ★ | ★ | ★ | ★ | - | ★ | ★ | ★ | 7 |
| Varwani_2021 | ★ | ★ | ★ | ★ | ★ | ★ | ★ | ★ | 8 |
| Xu_2021 | ★ | ★ | - | ★ | ★ | ★ | ★ | - | 6 |
| Zhang_2021 | ★ | ★ | ★ | ★ | ★ | ★ | ★ | - | 7 |
| Herald_2022 | ★ | ★ | ★ | ★ | ★ | ★ | ★ | ★ | 8 |
| Rahunathan_2022 | ★ | ★ | ★ | ★ | ★ | ★ | ★ | ★ | 8 |
| Zhang_2022 | ★ | ★ | ★ | ★ | ★ | ★ | ★ | ★ | 8 |
| Seiler_2023 | ★ | ★ | ★ | ★ | ★ | ★ | ★ | ★ | 8 |

**Supplemental Table S3: Quality assessment of included randomized controlled trials using the Cochrane risk of bias tool.**

| Author_Publication year | R | D | Mi | Me | S | O |
| --- | --- | --- | --- | --- | --- | --- |
| Abdelnabi_2021 | 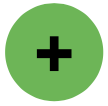 | 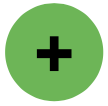 | 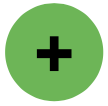 | 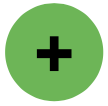 | 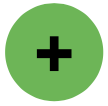 | 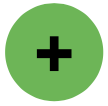 |
| Alcalai_2021 | 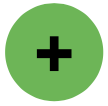 | 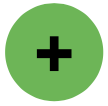 | 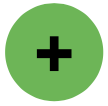 | 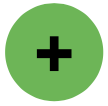 | 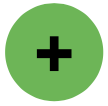 | 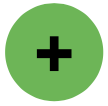 |
| W. Isa_2021 | 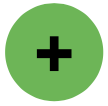 | 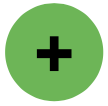 | 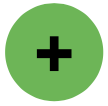 | 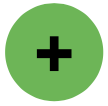 | 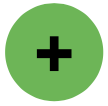 | 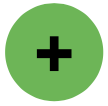 |

R: Bias arising from randomisation process

D: Bias due to deviations from intended interventions

Mi: Bias due to missing outcome data

Me: Bias in measurement of the outcome

S: Bias in selection of the reported result

O: Overall risk bias

**Supplemental Fig S1: The step-by-step procedure of trial sequential analysis in left ventricular (LV) thrombus resolution.**

**Supplemental Fig S2: PRISMA flowchart.**

**Supplemental Fig S3: TSA of DOACs and VKAs in *major bleeding*.**

**
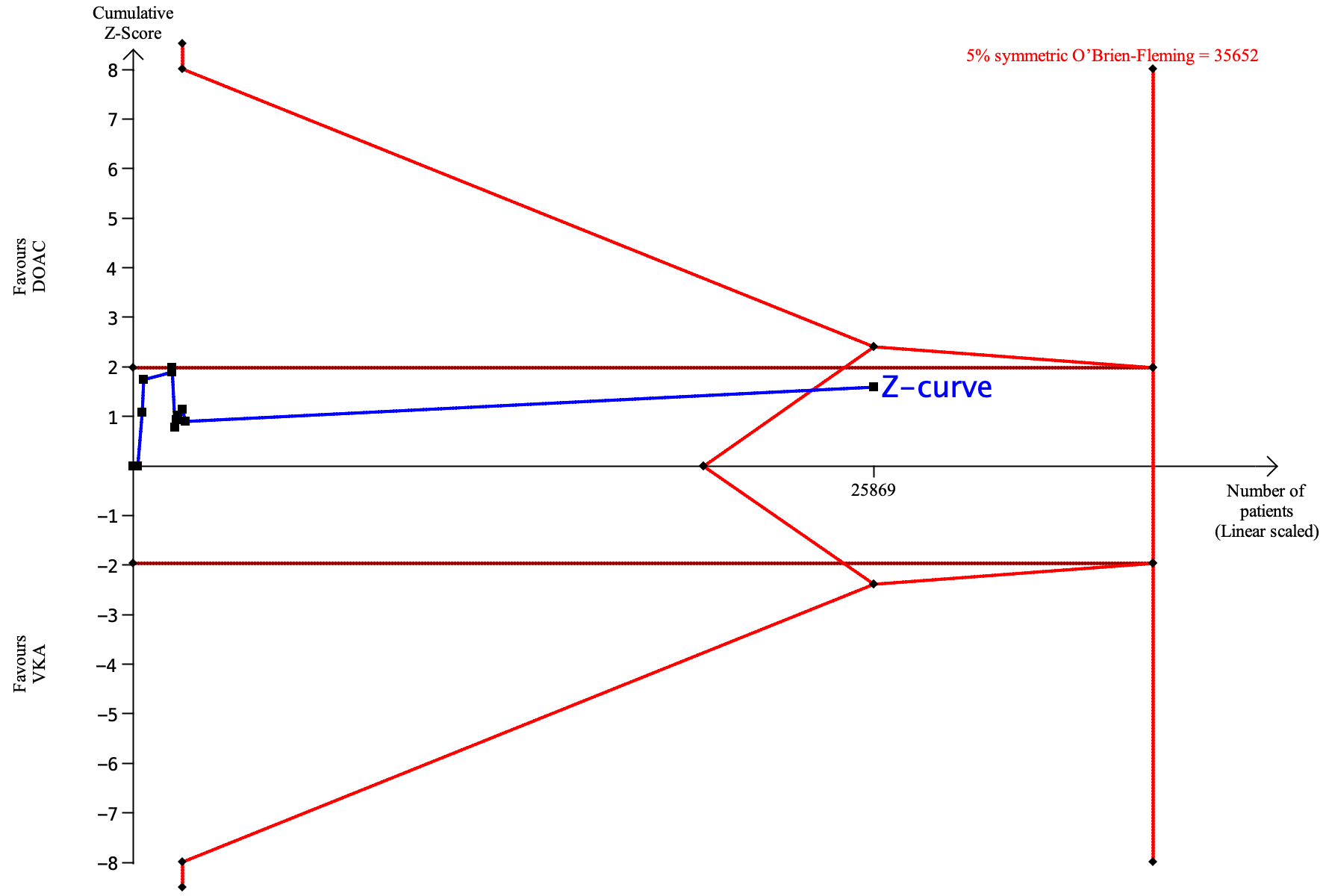
**

The cumulative Z-curve could not cross the superiority boundary no matter how many patients were added. When 24,000 patients were added, the boundary of futility was crossed before the superiority or traditional boundaries were crossed.

**Supplemental Fig S4: TSA of DOACs and VKAs in *major bleeding*.**

**
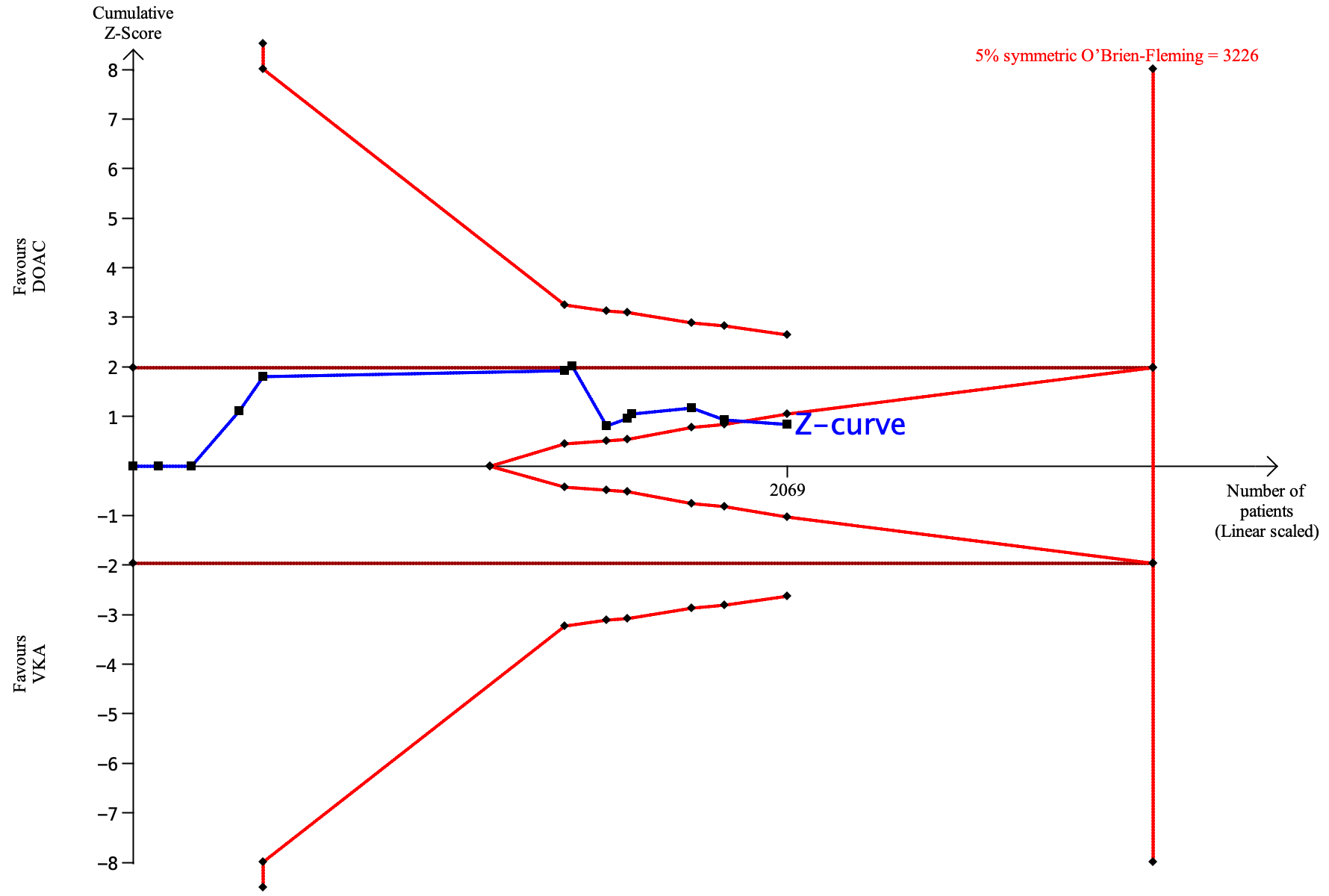
**

The cumulative Z-curve crossed the futility boundary at 2,069 patients, suggesting that an additional 200 patients are needed to demonstrate no difference in *major bleeding* between DOACs and VKAs.

**Supplemental Fig S5: TSA of DOACs and VKAs in *any bleeding*.**

**
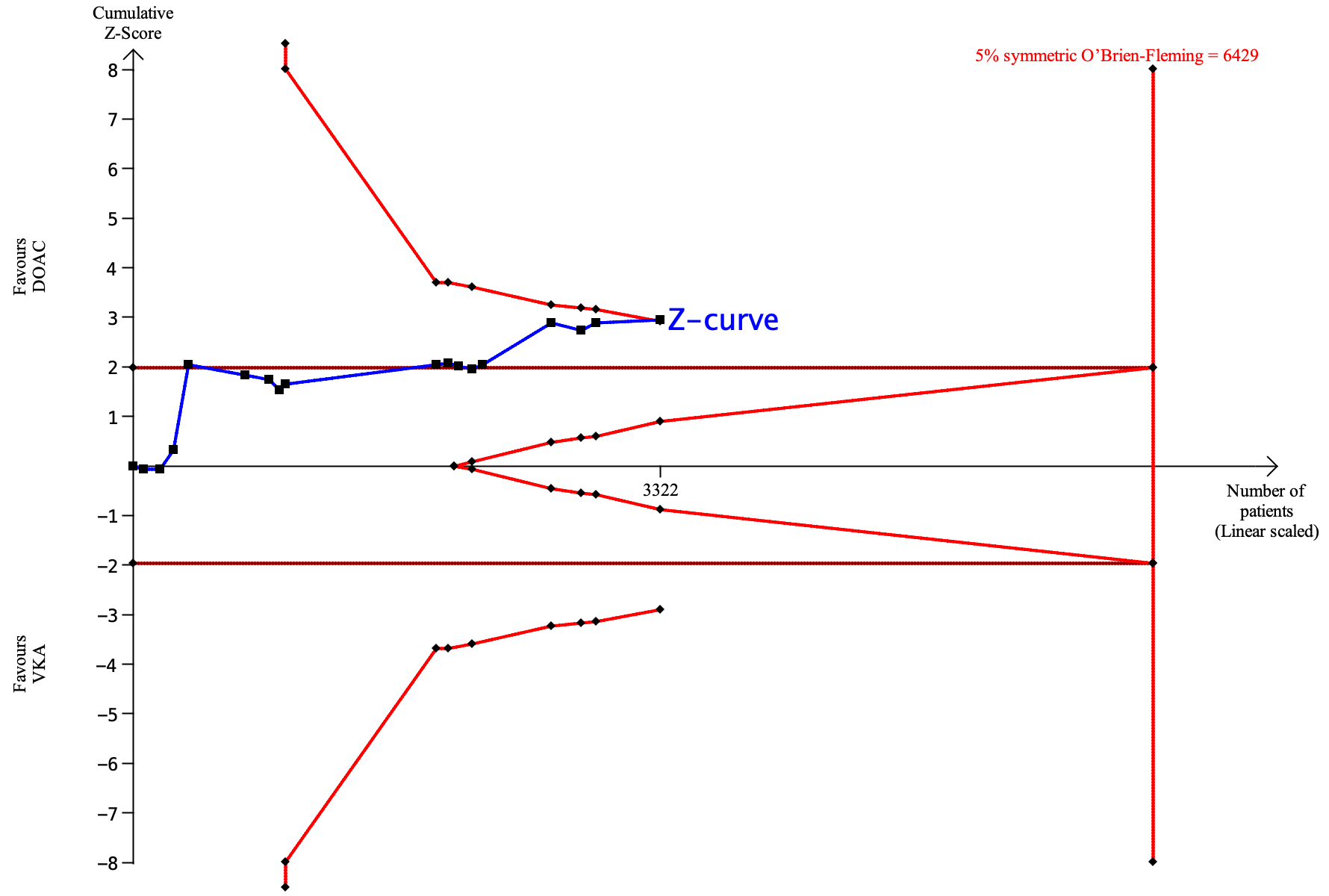
**

The cumulative Z-curve crossed the superiority boundary at 3,322 patients, suggesting that an additional 400 patients are needed to show that DOACs significantly reduce *any bleeding*.

**Supplemental Fig S6: TSA of DOACs and VKAs in *any bleeding*.**

**
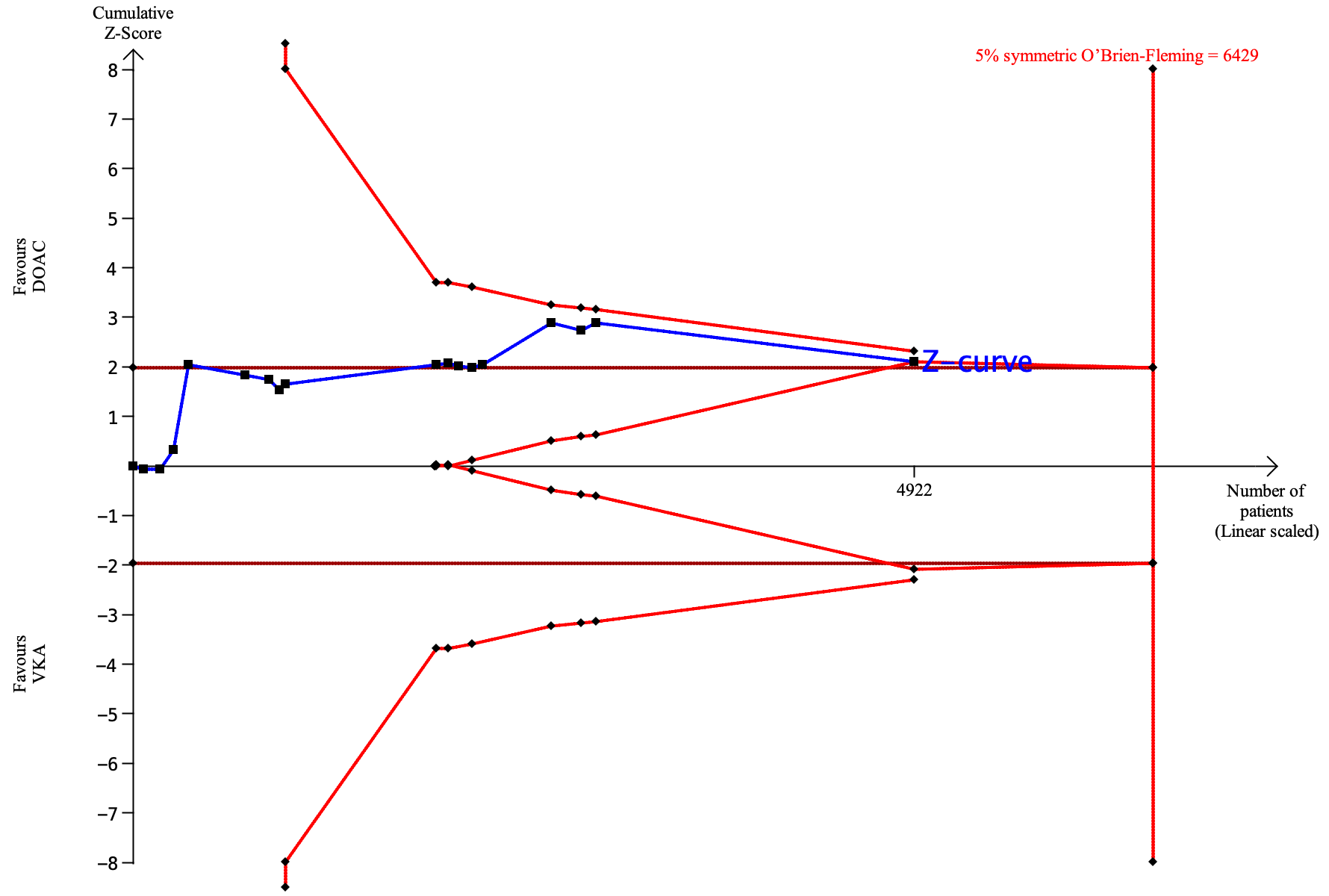
**

The cumulative Z-curve crossed the futility boundary at 4,922 patients, suggesting that an additional 2,000 patients are needed to prove absence of difference in any bleeding between DOACs and VKAs.
